# Comparison of self-rating of cognition and depression in patients with major depressive disorder

**DOI:** 10.1108/MIJ-02-2020-0005

**Published:** 2020-06-27

**Authors:** Kishen Berra, Charles Nguyen, Peter Bota

**Affiliations:** School of Biology, University of California Riverside, Riverside, California, USA; UC Riverside School of Medicine, Riverside, California, USA; College of Letters, Arts, and Sciences, University of California Berkeley, Berkeley, California, USA

**Keywords:** Major depressive disorder, CPFQ

## Abstract

**Purpose:**

The purpose of this paper is to discover if there is a correlation between scores on the Beck’s Depression Inventory (BDI) and the Cognitive and Physical Functioning Questionnaire (CPFQ) scores of 43 patients with major depression.

**Design/methodology/approach:**

In total, 43 adult patients with major depression were evaluated during their regularly scheduled outpatient appointment in a mental health clinic.

**Findings:**

There was an *R^2^* value of 0.6544 between the patients’ scores, a moderate-to-strong correlation which matches other observations that cognitive impairment increases in conjunction with severity of depression. This correlation lends further clinical support to the legitimacy of using the CPFQ as a simpler alternative to traditional neuropsychological testing, with further testing of the correlation between CPFQ and traditional neuropsychological testing results being a worthwhile potential field of study.

**Originality/value:**

Cognitive dysfunction is a frequent comorbidity in patients with depression, but while there is a brief and effective self- assessment for depression, the BDI, in common use, there is no equivalent test for cognitive dysfunction, and physicians are forced to rely on less accessible methods of neuropsychological testing.

## Introduction

Cognitive and executive dysfunctions are commonly present in patients with depression (Addington *et al.*, 2001). Cognition is defined as the mental process of acquiring knowledge and understanding through thought, experiences and the senses, and its impairment is pernicious to patient function and quality of life. Traditionally, clinicians have used neuropsychological tests to evaluate cognitive impairment in patients with major depression. However, these instruments are onerous to both clinicians and patients, limiting their accessibility for most patients (Fava *et al.*, 2009). Therefore, there is a clear need for a brief, self-rated assessment of cognitive function. The Massachusetts General Hospital Cognitive and Physical Functioning Questionnaire (CPFQ) was developed to assess seven common complaints of depressed patients regarding fatigue and cognitive problems. The CPFQ is a seven-item self-administered questionnaire with higher scores indicating impaired functioning, and it has been found to have strong internal consistency (Fava *et al.*, 2006). To our knowledge, this is the only self-rating scale for cognitive function currently in practice. However, there are several many self-rating scales for depression in use for many years. The Beck’s Depression Inventory (BDI), which assesses the severity of depression from a range of 0–63, is currently the most commonly used scale. We set out to assess patient responses to both scales and to determine the presence of a correlation between a patient’s scores on each of the scales. Our belief was that the CPFQ score would correlate with the BDI score, where the higher self-response scores on the CPFQ would relate to higher self-response scores on the Beck’s depression scale.

## Methods and materials

In total, 43 adult patients with major depression were evaluated during their regularly scheduled outpatient appointment in a mental health clinic. Diagnosis was based on their medical record, and these patients carried no other primary psychiatric disorders on Axis 1. Each was administered both the CPFQ and BDI. We collected patient’s age, sex and time since first diagnosis of major depression.

## Results

**Figure 1:**
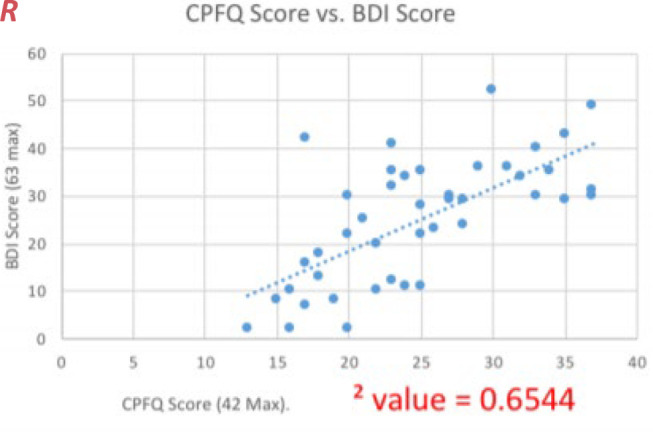
Graph of patient’s BDI and CPFQ scores

**Figure 2:**
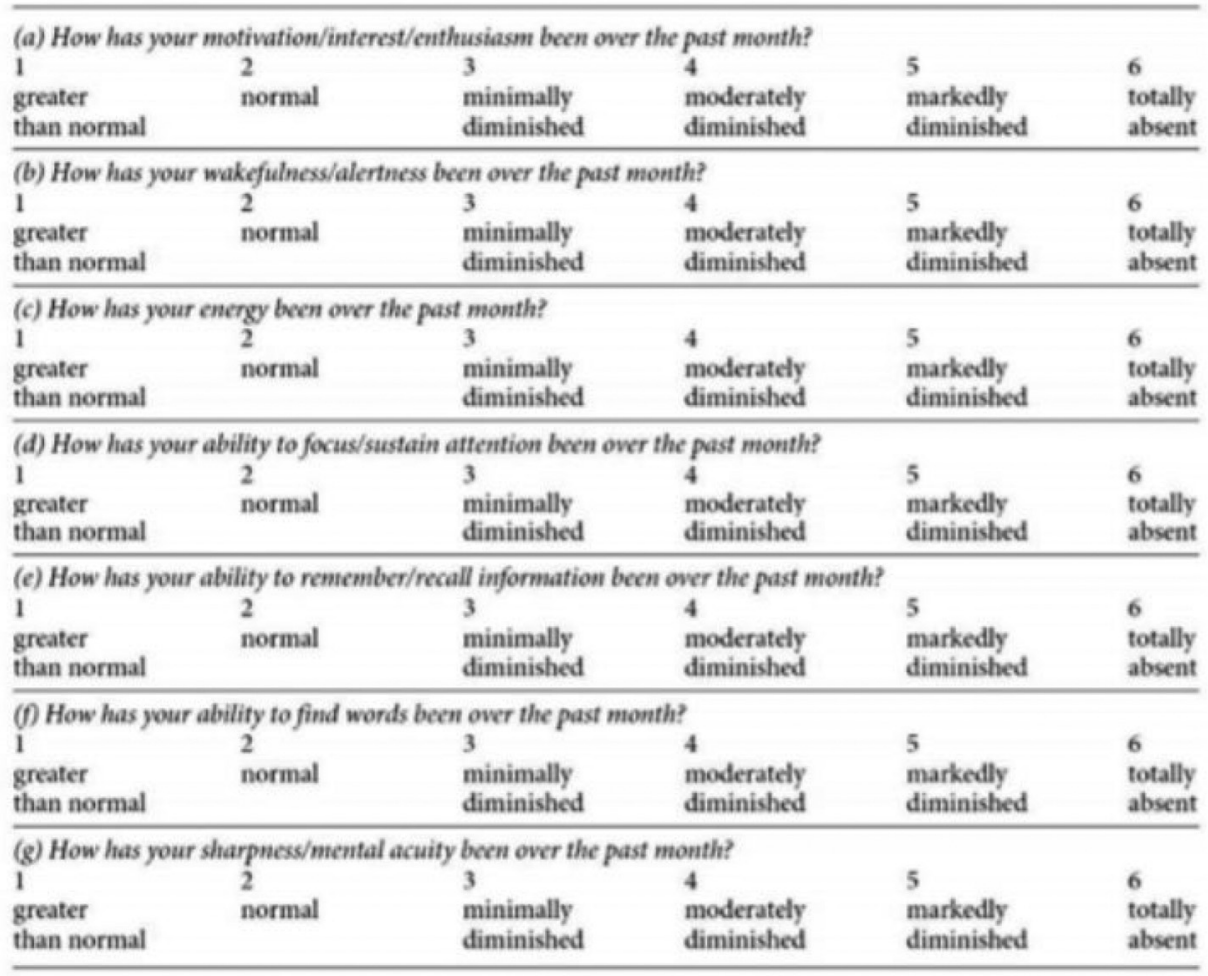
Sample CPFQ

**Figure 3:**
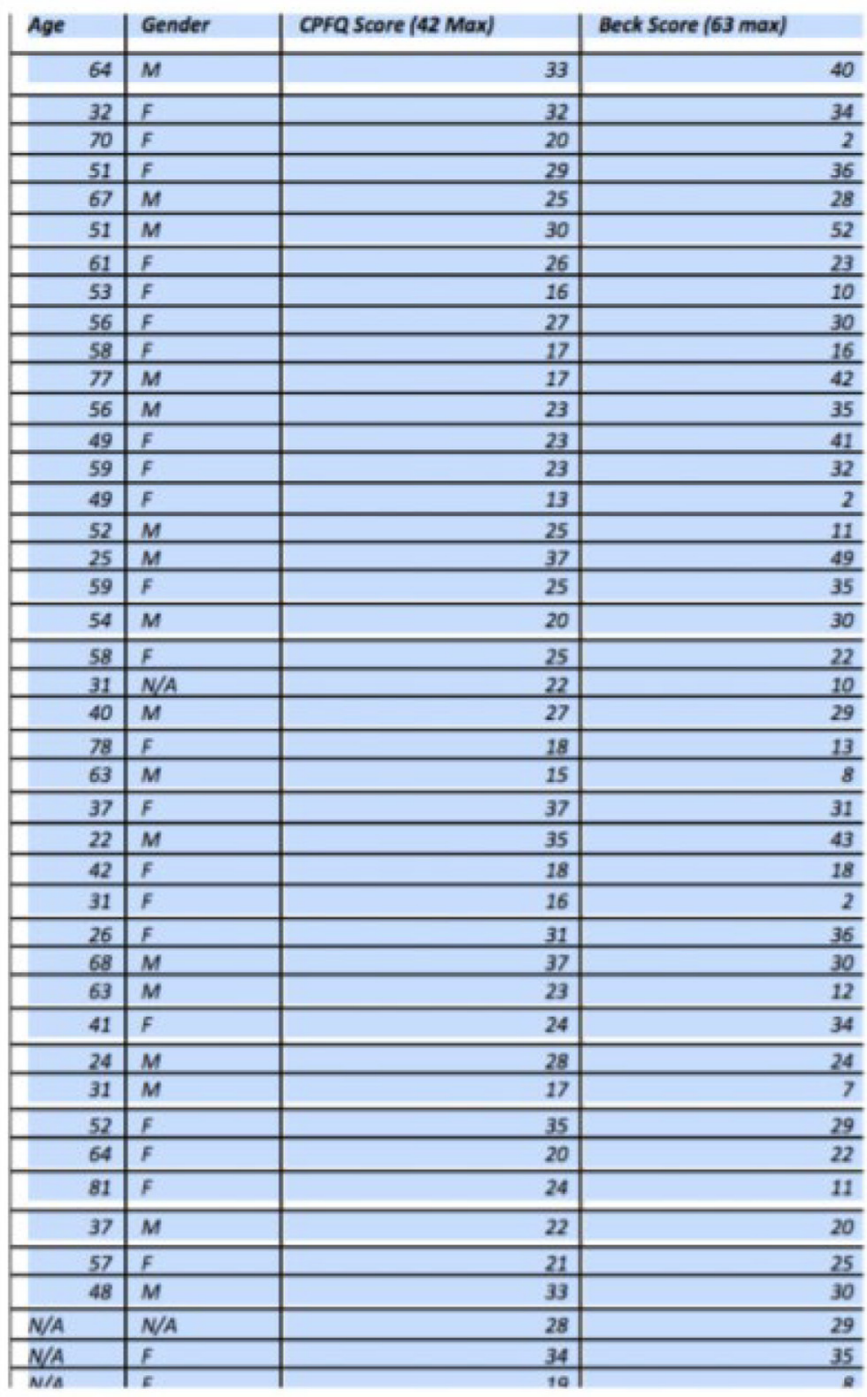
Patient data

There is a positive correlation between patient’s self-assessment of CPFG and BDI with an R-squared value of 0.6544. As the severity of depression increases (BDI), the cognitive impairment (CPFQ) also increases (Figures 1–3).

## Discussion

Cognitive impairment is emerging as an important therapeutic target in patients with psychiatric illnesses, including major depressive disorder. Based on the results we found, CPFQ was significantly correlated with the degree of depression, with a moderate to high correlation (r ∼ 0.654) indicating that the CPFQ is measuring a similar construct to the one measured by the BDI. Patients who scored higher on the BDI also tended to score higher on the CPFQ scale, which suggests that the more severe the depression, the greater the impairment of cognition (Fava *et al.*, 2009; Russo *et al.*, 2015). It is well known that major depressive disorder patients underperform when compared to healthy subjects in tasks measuring attention, executive functions and verbal learning, and our evidence also indicated that the more depressed the patient is, the worse their cognitive functions (Fava *et al.*, 2009; Russo *et al.*, 2015). Most importantly, clinicians will now be able to access cognition in a rapid fashion and determine if the treatment being prescribed is resulting in improved psychosocial functioning, which has been determined to be a more important marker than depression level for measuring patient outcomes (Fava *et al.*, 2009; Russo *et al.*, 2015), and cognitive dysfunction as a therapeutic target remains an area of significant study (Fava *et al.*, 2009). Appreciating that the CPFQ is not as comprehensive as traditional neuropsychological testing methods that assess cognition, it is still our hope that this tool may provide a snapshot for clinicians into patient cognitive status. In the future, it would be quite worthwhile to administer the CPFQ to patients who been administered neuropsychological testing and to compare the results. With our finding that there is a direct correlation between CPFQ score and BDI score, the CPFQ gains further clinical support, allowing clinicians to use this simple, user-friendly scale to assess the degree of cognitive impairment in their patients. As well, the current study focused on the uses for the CPFQ for determining cognitive impairment comorbid with depression, but further studies can and should be done on the merits of the CPFQ in determining the severity of cognitive impairment owing to other causes.

## Conclusions

This study demonstrates the potential of the CPFQ as an alternative to more time-intensive neuropsychological testing for determining the degree of cognitive impairment present in a patient by demonstrating a correlation with BDI scores that matches the correlation between depression severity and degree of cognitive impairment. As stated in the discussion section, these findings ought to be followed by further studies to broaden and corroborate the value of the CPFQ as a more accessible method of neuropsychological testing for cognitive impairment, which would ease and speed up the recognition and treatment of cognitive impairment, which has been recently recognized as a factor that severely damages quality of life and is therefore crucial to treat.

## References

[ref001] Addington A.M., Gallo J.J., Ford D.E. and Eaton W.W. (2001), “Epidemiology of unexplained fatigue and major depression in the community: the Baltimore ECA follow-up, 1981-1994”, Psychological Medicine, Vol. 31 No. 6, pp. 1037-1044. 11513371 10.1017/s0033291701004214

[ref002] Fava M., Graves L., Bennazzi F., Scalia M.J., Iosifescu D.V., Alpert J.E. and Papakostas G.I. (2006), “Prevalence of cognitive and physical adverse events during long term antidepressant treatment”, The Journal of Clinical Psychiatry, Vol. 67 No. 11, pp. 1754-1759. 17196056 10.4088/jcp.v67n1113

[ref003] Fava M., Iosifescu D.V., Pedrelli P. and Baer L. (2009), “Reliability and validity of the Massachusetts general hospital cognitive and physical functioning questionnaire”, Psychotherapy and Psychosomatics, Vol. 78 No. 2, pp. 91-97. 19218827 10.1159/000201934

[ref004] Russo M., Mahon K. and Burdick K. (2015), “Measuring cognitive function in MDD: emerging assessment tools”, Depression and Anxiety, Vol. 32 No. 4, pp. 262-269. 25421437 10.1002/da.22297 PMC4407945

